# Effects of physical exercise on outcomes of cardiac (dys)function in women with breast cancer undergoing anthracycline or trastuzumab treatment: study protocol for a systematic review

**DOI:** 10.1186/s13643-019-1154-x

**Published:** 2019-10-24

**Authors:** Pedro Antunes, Dulce Esteves, Célia Nunes, Anabela Amarelo, José Fonseca-Moutinho, Vera Afreixo, Henrique Costa, Alberto Alves, Ana Joaquim

**Affiliations:** 1 Research Center in Sport Sciences, Health and Human Development (CIDESD) & Sport Sciences Department, Universidade da Beira Interior, Covilhã, Portugal; 2 Associação de Cuidados de Suporte em Oncologia, Sanfins, Portugal; 3 Mathematics Department, Universidade da Beira Interior, Covilhã, Portugal; 4 Oncology Department, Centro Hospitalar Vila Nova de Gaia/Espinho, Vila Nova Gaia, Portugal; 5 Health Sciences Department, Universidade da Beira Interior, Covilhã, Portugal; 6 CIDMA - Center for Research and Development in Mathematics and Applications, iBiMED - Institute for Biomedicine, Department of Mathematics, Universidade de Aveiro, Aveiro, Portugal; 7 Psychiatry and Mental Health Department, Centro Hospitalar de Setúbal, Setúbal, Portugal; 8 Research Center in Sports Sciences, Health Sciences and Human Development (CIDESD) & Instituto Universitário da Maia, Maia, Portugal

**Keywords:** Breast cancer, Exercise, Cardiotoxicity, Cardiovascular function, Systematic review

## Abstract

**Background:**

Cardiotoxicity is a known complication and one of the most adverse effects from the use of conventional treatments such as anthracyclines and trastuzumab in breast cancer (BC) care. This phenomenon has been associated with the restriction of therapeutic options and the increase of cardiovascular complications, which may compromise the survival of patients. Implementation of preventive strategies is an important approach for the management of this issue. Physical exercise has been proposed as a non-pharmacological strategy to counteracting cardiotoxicity. The aim of this protocol is to describe the rationale and methods for a systematic review of published randomized controlled trials (RCTs) that have analysed the effects of physical exercise on outcomes of cardiac (dys)function in women with BC undergoing neoadjuvant or adjuvant treatment containing anthracyclines and/or trastuzumab.

**Methods and analysis:**

This is a protocol for a systematic review reported according to the PRISMA-P 2015 checklist. Randomized controlled trials (RCTs) will be included. The literature will be screened on MEDLINE, EMBASE, the Cochrane Central Register of Controlled Trials, ISI Web of Science and Scopus. The risk of bias of the included RCTs will be assessed using the Cochrane Collaboration’s tool. The primary outcomes will be systolic function (left ventricular ejection fraction), diastolic function (E/A’ ratio, deceleration time of early left ventricular filling, isovolumetric relaxation time, E/E’ septal and lateral ratio) and myocardial deformation imaging outcomes (strain and strain rate [measured in longitudinal, radial, or circumferential directions]). Secondary outcomes will be cardiac biomarkers (troponin I or T, high-sensitivity troponin I or T, brain natriuretic peptide, amino terminal of B-type natriuretic peptide). Data will be descriptively reported, and quantitative synthesis will also be considered if the included studies are sufficiently homogenous.

**Discussion:**

This systematic review will help to understand the effectiveness of physical exercise on counteracting cardiotoxicity related to anticancer therapies in women with BC.

**Systematic review registration:**

PROSPERO CRD42018096060.

## BACKGROUND

Breast cancer (BC) is the most diagnosed malignancy and the major cause of cancer-related death in the female gender [1]. Over the past two decades, due to preventive actions, early screening and advances in anticancer therapies, the BC management has been marked by a notable progress reflected by the improvement in survival rates [2]. However, despite the optimistic prospects in the fight against BC, survivorship is often marked by several treatment-related side effects and poor quality of life [3, 4]. Notably, cardiotoxicity has been recognized as a serious issue in clinical practice, restricting treatment options and contributing to morbidity and mortality among BC patients [5, 6].

Cardiac dysfunction, which involves direct effects of the treatment on heart function and structure [7], is a well-established side effect from the use of conventional cardiotoxic drugs such as anthracyclines (the cornerstone of BC chemotherapy) and trastuzumab (the standard of care for the treatment of human epidermal growth factor receptor 2 (HER2)-positive) in BC treatment [8]. Anthracycline-induced cardiotoxicity is mostly influenced by cumulative dose, leading to an irreversible cardiac damage (type I cardiotoxicity) [9]. The anthracycline-related cardiac effects are commonly manifested by asymptomatic or symptomatic left ventricular dysfunction leading to heart failure, myocardial ischemia, arrhythmias, hypertension, myocarditis, pericarditis and thromboembolism [10]. On the other hand, trastuzumab-related cardiotoxicity is not dose-dependent and is often reversible (type II cardiotoxicity) after treatment discontinuation [11]. Nevertheless, the elevated incidence of cardiac dysfunction in patients treated with trastuzumab after anthracyclines is a growing concern [12].

According to the American Society of Echocardiography and the European Association of Cardiovascular Imaging, cardiotoxicity is defined by a decrease in left ventricular ejection fraction (LVEF) of > 10% to a value of < 53% [13]. Currently, serial determination of LVEF is the standard marker for reporting cardiotoxicity related to anticancer therapies [7]. However, the determination of LVEF has substantial limitations and is considered a poor sensitivity parameter to detect cardiotoxicity at an early stage [14]. As such, an integrated approach that includes the assessment of echocardiographic parameters (diastolic function and myocardial deformation imaging) and biomarkers of cardiac injury has been recommended for a better monitoring of cardiac (dys)function [13, 15]. Besides the importance of screening, the implementation of preventive strategies that optimize cardiovascular care in patients with BC treated with cardiotoxic agents is also an important component to avoid this issue, but currently, they are limited [16].

Physical exercise is recognized as a viable non-pharmacological approach for the management of several cardiovascular risk factors [17, 18] and as a safe and effective supportive care for cancer survivors [19]. Evidence from meta-analysis highlights its beneficial effects on physiological and psychological outcomes, during [20] or after treatment [21], as well as its important role in surveillance and disease recurrence [22]. Furthermore, exercise has also been proposed as a promising strategy to prevent cardiotoxicity related to anticancer therapies [7, 23]. There are several review studies that analysed and described the potential protection mechanisms of physical exercise against cardiotoxicity, but most of these have reported data from animal studies [24–27]. To the best of our knowledge, no systematic review has summarized the effects of physical exercise on cardiac (dys)function induced by neoadjuvant or adjuvant treatment containing anthracyclines and/or trastuzumab in adult women with BC.

Therefore, this systematic review will aim to analyse the existing evidence regarding the role of physical exercise on outcomes of cardiac (dys)function in intervention studies that involved women with BC who were undergoing neoadjuvant or adjuvant treatment containing anthracyclines and/or trastuzumab.

## Methods and analysis

### Study design

This is a protocol for a systematic review which is registered in the international Prospective Register of Systematic Reviews (PROSPERO; registration number: CRD42018096060) and has been developed in accordance with the Preferred Reporting Items for Systematic Review and Meta-Analysis Protocols (PRISMA-P) [28, 29] (available in Additional file 1).

### Patient and public involvement

No patients will be involved in this study.

#### Eligibility criteria

We will include studies that meet the following criteria:

##### Study type

Published randomized controlled trials (RCTs) will be eligible. Due to funding restraints, we will include studies in English, French, Germany, Portuguese and Spanish language.

##### Participants

Studies that involving adult women (> 18 years old) with BC, who were undergoing in a neoadjuvant or adjuvant treatment containing anthracyclines and/or trastuzumab and performed concurrently a physical exercise intervention, were included. Studies which involved other cancer types beyond BC will be excluded.

##### Intervention

Studies that involved aerobic training (any exercise form that uses large muscle groups which predominately stresses the cardiovascular system, such as walking, jogging, cycling) and resistance training (any exercise form that requires a muscle or a muscle group to work against external resistance which predominately stresses the musculoskeletal system, such as squats, chest press), according to the American College of Sports Medicine [30], isolated or in combination, will be included. Trials will not be considered if:

- Yoga, tai chi chuan, qigong or pilates was defined as physical intervention.
- The physical exercise intervention group performed other supportive care (e.g. dietary plan).

##### Comparator(s)

Comparators will include non-exercise group (i.e. waiting list, control or placebo).

##### Outcomes

Studies that report the absolute and/or percentage change from baseline to the end of the intervention and/or at follow-up, on at least one of the following outcomes, will be considered:

- Primary outcomes: resting LVEF
- Secondary outcomes: resting parameters of diastolic function [E/A’ ratio; deceleration time of early left ventricular filling (DT); isovolumetric relaxation time (IVRT); E/E’ lateral and E/E’ septal]; resting parameters of myocardial deformation imaging (strain and strain rate); cardiac biomarkers (troponin I or T; high-sensitivity troponin I or T; brain natriuretic peptide; amino terminal of B-type natriuretic peptide)

#### Search methodology for identification of studies

##### Electronic databases

Literature search will be performed in the following electronic bibliographic databases: MEDLINE (via PubMed), EMBASE (Via embase.com), the Cochrane Central Register of Controlled Trials (CENTRAL), ISI Web of Science and Scopus. Moreover, clinical trial registers such as ClinicalTrials.gov (https://ClinicalTrials.gov), World Health Organization (WHO) trials portal (www.who.int/ictrp/en/) and International Clinical Trials Registry Platform registry (http://www.isrctn.com) will be also scanned to identify ongoing and/or protocols trials. Furthermore, the references of the included manuscripts and relevant reviews will be checked. No restriction will be applied regarding the year of publication and the language of the study. This researching process will be independently conducted by two review authors (PA and DE).

##### Search terms and keywords

The specific search strategies were created through a consensus between all the authors and will include the following four concepts and their related terms: (1) condiction (i.e. breast cancer), (2) cancer treatment (i.e. anthracyclines and trastuzumab), (3) exercise (i.e. aerobic exercise and resistance exercise) and (4) study type (i.e. controlled trial). It will be used as text terms and indexing terms from the thesaurus of the databases (i.e. Medical Subject Headings (MeSH) for MEDLINE and CENTRAL and Emtree for EMBASE) and Boolean operators. The search strategy for MEDLINE is outlined at Table 1 and will be adapted for the further databases. This researching process will be independently conducted by two review authors (PA and DE).

**Table 1:** Search strategy for MEDLINE (via PubMed)

Concept	Related terms
#1 condition	((breast neoplasms[MeSH]) OR ((breast[MeSH] OR breast diseases[MeSH]) AND neoplasms[MeSH])) OR ((Breast[tiab] OR mammary[tiab]) AND cancer*[tiab] OR carcinoma*[tiab] OR tumour*[tiab] OR malignant*[tiab] OR neoplasm*[tiab]))
#2 cancer treatment	((chemotherapy[MeSH]) OR anthracycline[MeSH]) OR trastuzumab[MeSH]) OR Antibodies, Monoclonal, Humanized[MeSH]) OR (chemotherap*[tiab] OR anthracycline*[tiab] OR trastuzumab[tiab] OR herceptin[tiab]))
#3 exercise	((Exercise training[MeSH]) OR Resistance training[MeSH]) OR Exercise therapy[MeSH]) OR ((Exercise[tiab] OR training[tiab] OR sport*[tiab]) AND aerobic*[tiab] OR resistance*[tiab] OR strength*[tiab] OR weight [tiab] OR endurance*[tiab]))
#4 study type	((randomized controlled trial[pt]) OR controlled clinical trial[pt]) OR (randomized[tiab] OR randomly[tiab] OR trial[tiab]))

#1 AND #2 AND #3 AND #4

#### Data management

##### Studies selection

Screening search results will be entered into the latest version of EndNote (Clarivate Analytics) and duplicates will be removed. These procedures will be independently conducted by two review authors (PA and DE). After this, to test the eligibility criteria, the same two review authors will independently evaluate the title and abstract of the studies. The manuscripts that appear to meet the eligibility criteria will be recorded using an Excel predesigned criterion data collection form. Then, the same two review authors (PA and DE) will meet in person and perform a non-blind full-text screening to take a final inclusion decision. Reasons for exclusion will be recorded. Possible disagreements will be resolved by discussion or, if necessary, by the judgement of a third author (AAl). Whenever required, the study’s corresponding authors will be contacted to provide possible ambiguous or lack of necessary information.

#### Data extraction

Two review authors (CN and VA) will independently perform data extraction using a Microsoft Office Excel version 2016 (Microsoft Corporation, Redmond, WA, USA) predesigned criterion data collection form. The following data will be collected:

- Study characteristics: primary aim, authors, year of publication, journal name, country and sample size
- Participant characteristics: mean age, disease stage and treatment scheme
- Intervention characteristics: exercise type, intensity, frequency, time, supervision, location and, if provided, we will also highlight safety and feasibility
- Comparison details: placebo or control group
- Interested study outcomes: data of the study outcomes (effect size, 95% CI, standard mean deviation) and its measuring methods

When ambiguous or clarification of data is required, the study’s corresponding authors will be contacted (via email). Possible disagreements during this process will be resolved by consensus or, if necessary, by a third review author’s judgement (AAl).

#### Assessment risk of bias of the included studies

Two review authors (AJ and AA) will independently assess and score the methodological quality of the included trials using the Cochrane Collaboration’s tool for assessing risk of bias, according to the following domains: random sequence generation, allocation concealment, blinding of participants and personnel, blinding of outcome assessment, incomplete outcome data, selective reporting and other potential sources of bias [31]. The level of risk of bias will be determined for each domain: (1) high risk, (2) unclear risk or (3) low risk. Possible disagreements shall be resolved by discussion and, if necessary, by consulting a third review author (AAl). We will use the Grades of Recommendation, Assessment, Development and Evaluation (GRADE) approach to assess the quality of the evidence across studies [32].

#### Heterogeneity assessment

Statistical heterogeneity will be tested by the χ^2^ test and quantified by the *I*^2^ statistic. Heterogeneity will be assumed if we verified a *p* value of < 0.1 for χ^2^ and *I*^2^ of ≥ 50% [33]. We will perform sensitivity analyses stratified according to the risk of bias.

#### Data synthesis

Data will be managed using the Review Manager software version 5.3 (Nordic Cochrane Centre, the Cochrane Collaboration, Copenhagen, Denmark). The characteristics and findings extracted from the included studies will be exposed in a descriptive way, through tables, and complemented with a synthetic narrative which explores the relationship between the trials. If heterogeneity is found in most studies, regarding design and comparator, random effects model meta-analyses will be performed. For continuous outcomes, we planned to estimate standardized mean difference (SMD) with confidence interval of 95% (CI 95%). When standard deviations are not available, they will be calculated from standard errors, CI or *t* values for both groups for each study included. The magnitude of the effect size will be reported according to Cohen’s classification with the following: small (SMD = 0.2–0.5), moderate (SMD = 0.5–0.8) and large (SMD > 0.8) [34].

#### Assessment of reporting bias

We will examine funnel plots corresponding to meta-analysis of the primary outcomes to assess the potential for small-study effects such as publication bias if we include more than 10 studies in an analysis.

#### Subgroups analyses

If sufficient data is available, we will perform subgroup analyses according to the following:

- Intervention type (aerobic or resistance, concomitant or isolated)
- Exercise intensity (light, moderate, vigorous)
- Treatment scheme (including anthracyclines or trastuzumab, concomitant or isolated)

#### Ethics and dissemination

Ethics approval is not required because this is a protocol for a systematic review not involving personal data or the exercise of any intervention in patients. The findings of this study will be submitted to a peer-reviewed journal for publication, will be presented at relevant conferences and will be also part of the main author’s PhD thesis.

## Discussion

The notable progress in the care of BC has led to a marked improvement in survival rates [2]. Despite the optimistic prospects in the fight against BC, these patients face several treatment-related side effects [3, 4]. Cardiac dysfunction has been recognized as a major concern from the use of conventional cardiotoxic drugs [6], such as anthracyclines and trastuzumab, which can occur either during treatment or after it [7]. The implementation of preventive strategies to optimize and balance cardiac health is needed. Physical exercise has been emerging as a potential approach for counteracting cardiotoxicity related to anticancer therapies [7, 23]. Previous review studies have analysed and described the potential protection mechanisms of physical exercise against cardiotoxicity [24–27], but most of these have reported data from animal studies. Until now, no systematic review has summarized the effects of physical exercise on cardiac (dys)function induced by neoadjuvant or adjuvant treatment containing anthracyclines and/or trastuzumab in adult women with BC.

## Limitations

We anticipate some limitations. There may be a risk of bias as we only include published articles. Moreover, this systematic review may be limited by the lack of studies and significant heterogeneity among them.

## Conclusion

In this systematic review, we will conduct a comprehensive and rigorous research to summarize and clarify the potential cardioprotective effect of physical exercise at mitigating cardiotoxicity in women with BC undergoing neoadjuvant or adjuvant treatment containing anthracyclines and/or trastuzumab. We expect to present solid findings in this work that it may facilitate the integration of future policies which aim at balancing the negative cardiac effects related to the use of cardiotoxic drugs in the care management of BC, making decisions regarding the practice of exercise.

## Supplementary information

**Additional file 1.** PRISMA-P 2015 Checklist.

## Data Availability

The findings of this review will be related and extracted from published papers with easy access.

## Abbreviations

BC: Breast cancer
CENTRAL: Cochrane Central Register of Controlled Trials
GRADE: Grading of Recommendations Assessment, Development and Evaluation
LVEF: Left ventricular ejection fraction
PRISMA-P: Preferred Reporting Items for Systematic Review and Meta-Analysis Protocols
PROSPERO: Prospective Register of Systematic Reviews
WHO: World Health Organization
