# Supplementary material for: Effects of physical exercise on outcomes of cardiac (dys)function in women with breast cancer undergoing anthracycline or trastuzumab treatment: study protocol for a systematic review
Source: Syst Rev. 2019 Oct 24;8:239. doi: 10.1186/s13643-019-1154-x (PMC6813963; doi:10.1186/s13643-019-1154-x)
Supplement: Supplementary file 1 — Additional file 1. PRISMA-P 2015 Checklist. [file 13643_2019_1154_MOESM1_ESM.pdf]

# PRISMA-P 2015 Checklist

**Manuscript Title**

Effects of physical exercise on outcomes of cardiac (dys)function in women with breast cancer undergoing anthracycline-containing or trastuzumab: study protocol for a systematic review.

***Corresponding author**

Pedro Miguel da Silva Antunes

Research Center in Sport Sciences, Health and Human Development (CIDESD), Department of Sport Sciences, University of Beira Interior, Covilhã Portugal, Rua Mateus Fernandes Lote 5 nº 37 1º C, pantunes_14@hotmail.com, 969270946

| Section/topic | # | Checklist item | Information reported: Yes | Information reported: No | Line number(s) |
|---|---|---|---|---|---|
| **ADMINISTRATIVE INFORMATION** | | | | | |
| **Title** | | | | | |
| Identification | 1a | Identify the report as a protocol of a systematic review | X☐ | ☐ | Page 1, line 1-3 |
| Update | 1b | If the protocol is for an update of a previous systematic review, identify as such | ☐ | X☐ | ---------- |
| **Registration** | 2 | If registered, provide the name of the registry (e.g., PROSPERO) and registration number in the Abstract | X☐ | ☐ | Page 4, line 80<br>Page 7, line 152-153 |
| **Authors** | | | | | |

BioMed Central
The Open Access Publisher

| Section/topic | # | Checklist item | Information reported Yes | No | Line number(s) |
|---|---|---|---|---|---|
| Contact | 3a | Provide name, institutional affiliation, and e-mail address of all protocol authors; provide physical mailing address of corresponding author | X☐ | ☐ | Page 1-2, line 5-34 |
| Contributions | 3b | Describe contributions of protocol authors and identify the guarantor of the review | X☐ | ☐ | Page 15-16, line 346-351 |
| **Amendments** | 4 | If the protocol represents an amendment of a previously completed or published protocol, identify as such and list changes; otherwise, state plan for documenting important protocol amendments | ☐ | X☐ | ---------- |
| **Support** | | | | | |
| Sources | 5a | Indicate sources of financial or other support for the review | X☐ | ☐ | Page 15, line 339-343 |
| Sponsor | 5b | Provide name for the review funder and/or sponsor | X☐ | ☐ | Page 15, line 339-343 |
| Role of sponsor/funder | 5c | Describe roles of funder(s), sponsor(s), and/or institution(s), if any, in developing the protocol | X☐ | ☐ | Page 15, line 339-343 |
| **INTRODUCTION** | | | | | |
| **Rationale** | 6 | Describe the rationale for the review in the context of what is already known | X☐ | ☐ | Page 5-6, line 69-147 |
| **Objectives** | 7 | Provide an explicit statement of the question(s) the review will address with reference to participants, interventions, comparators, and outcomes (PICO) | X☐ | ☐ | Page 5, line 100-116 |
| **METHODS** | | | | | |
| **Eligibility criteria** | 8 | Specify the study characteristics (e.g., PICO, study design, setting, time frame) and report characteristics (e.g., years considered, language, publication status) to be used as criteria for eligibility for the review | X☐ | ☐ | Page 7-8, line160-193 |
| **Information sources** | 9 | Describe all intended information sources (e.g., electronic databases, contact with study authors, trial registers, or other grey literature sources) with planned dates of coverage | X☐ | ☐ | Page 8, line208-217 |
| **Search strategy** | 10 | Present draft of search strategy to be used for at least one electronic database, including planned limits, such that it could be repeated | X☐ | ☐ | Page 8-9, line 218 |
| ***STUDY RECORDS*** | | | | | |
| Data management | 11a | Describe the mechanism(s) that will be used to manage records and data throughout the review | X☐ | ☐ | Page 10-11, line 220-236 |

| Section/topic | # | Checklist item | Information reported: Yes | Information reported: No | Line number(s) |
|---|---|---|---|---|---|
| Selection process | 11b | State the process that will be used for selecting studies (e.g., two independent reviewers) through each phase of the review (i.e., screening, eligibility, and inclusion in meta-analysis) | X☐ | ☐ | Page 10-11, line 220-236 |
| Data collection process | 11c | Describe planned method of extracting data from reports (e.g., piloting forms, done independently, in duplicate), any processes for obtaining and confirming data from investigators | X☐ | ☐ | Page 10-11, line 220-236 |
| **Data items** | 12 | List and define all variables for which data will be sought (e.g., PICO items, funding sources), any pre-planned data assumptions and simplifications | X☐ | ☐ | Page 11-12, line 206-219 |
| **Outcomes and prioritization** | 13 | List and define all outcomes for which data will be sought, including prioritization of main and additional outcomes, with rationale | X☐ | ☐ | Page 7, line 152-162 |
| **Risk of bias in individual studies** | 14 | Describe anticipated methods for assessing risk of bias of individual studies, including whether this will be done at the outcome or study level, or both; state how this information will be used in data synthesis | X☐ | ☐ | Page 11, line 238-252 |
| ***DATA*** | | | | | |
| **Synthesis** | 15a | Describe criteria under which study data will be quantitatively synthesized | X☐ | ☐ | Page 12-13, line 269-281 |
| | 15b | If data are appropriate for quantitative synthesis, describe planned summary measures, methods of handling data, and methods of combining data from studies, including any planned exploration of consistency (e.g., $I^2$, Kendall's tau) | X☐ | ☐ | Page 12-13, line 269-281 |
| | 15c | Describe any proposed additional analyses (e.g., sensitivity or subgroup analyses, meta-regression) | X☐ | ☐ | Page 13, line 288-293 |
| | 15d | If quantitative synthesis is not appropriate, describe the type of summary planned | X☐ | ☐ | Page 13, line 273-275 |
| **Meta-bias(es)** | 16 | Specify any planned assessment of meta-bias(es) (e.g., publication bias across studies, selective reporting within studies) | X☐ | ☐ | Page 12, line 265-267 |
| **Confidence in cumulative evidence** | 17 | Describe how the strength of the body of evidence will be assessed (e.g., GRADE) | X☐ | ☐ | Page 13, line 280-281 |
